# Using behavioural and social sciences to inform public policies during COVID-19, Uruguay

**DOI:** 10.2471/BLT.21.287071

**Published:** 2021-09-28

**Authors:** Alejandra López Gómez, Denisse Dogmanas, Nicolas Brunet-Adami, Nicolas Bagattini, Ricardo Bernardi

**Affiliations:** a Universidad de la República, Tristán Narvaja 1674, CP 11400, Montevideo, Uruguay.; b Centro Uno, Montevideo, Uruguay.; c Academia Nacional de Medicina, Montevideo, Uruguay.

The coronavirus disease 2019 (COVID-19) pandemic has challenged research capacities at global and national levels. The scientific community has played an important role in informing public policies for the management of the pandemic and has been vital to the translation of knowledge into actions and interventions. The relationship between scientific knowledge and public policies has been especially critical in low- and middle-income countries that have less developed local research capacities^1^ and therefore low impact. 

Human behaviours are at the centre of viral transmission^2^ and are relevant to changing the epidemiological dynamics of the virus through effective non-pharmaceutical interventions.^3^ However, understanding complex and multidimensional issues such as human behaviour requires socioecological and transdisciplinary perspectives.^4^

Behavioural and social science theories and models are useful to understand why, what and how people can change their behaviour in critical situations such as the COVID-19 pandemic.^5^ These theories and models provide insights into how behaviours, thoughts, attitudes and emotions are shaped in specific social contexts. Understanding the link between epidemiological dynamics and socioeconomic inequalities is especially important to predict protective behaviours. Additionally, the effects of COVID-19 interact with the psychological characteristics of the population. The pandemic’s dynamics affect and are affected by mental health conditions of the population^6^ and may alter social behavioural response patterns. Analysing the links between social and individual factors requires the production of quality and real-time data with a comprehensive approach. 

Here we describe the experience of Uruguay in generating evidence of behavioural dynamics and psychological effects of COVID-19 to inform decision-making in the country.

During 2020, Uruguay was considered a success in the management of the pandemic due to the low number of infections, severe cases and COVID-19 related deaths. One of the factors behind this success was the government’s establishment of the Honorary Scientific Advisory Group in April 2020, a group composed of experts from biomedical, health and data sciences. The group’s purpose was to generate systematic evidence to inform and advise on public policy for the management of the pandemic.^7^ In December 2020, the first wave of the pandemic started in Uruguay. The advisory group warned of the need to consider behavioural science inputs to understand current social interactions. To meet this objective, the group recommended the creation of a Socioeconomic and Behavioural Observatory to produce and disseminate knowledge on the behavioural dynamics and socioeconomic and mental health effects of COVID-19 on the Uruguayan population. The observatory’s purpose is to provide insight into the design and implementation of evidence-based interventions and public communication campaigns. The observatory thus became a platform for the generation of continuous, real-time and quality evidence.

The observatory facilitated joint research from an interdisciplinary team of behavioural, social and health sciences researchers from different academic institutions. The United Nations Development Programme supported the continuous collection and dissemination of data.

Since February 2021, the observatory has conducted a longitudinal study of nine waves using a panel of a statistically nationally representative sample of the population aged 18 years and older (*n* = 400). Responses were collected through an automated, auto-administrated questionnaire delivered via WhatsApp and followed up through phone calls if no response was received. To minimize respondent fatigue bias, brief questionnaires of up to 12 questions were used in the measurements. This methodological design enabled the examination of the population’s COVID-19 risk perceptions, predictors of risk behaviour, perceptions of effectiveness and adherence to non-pharmaceutical interventions and mental health effects.^8^ The selection of these subjects was based on the available international evidence as well as the local relevance of this information for the management of the pandemic. Obtaining data that illustrated better ways to promote and encourage the adoption of protective health behaviours and effective interventions was crucial. Several international studies have indicated that both behaviour and adherence to non-pharmaceutical interventions are influenced by risk perception.^9^ The first observatory study took place during February 2021 and aimed to assess the public’s risk perception of COVID-19 – considered as a psychological construct including social, emotional, cognitive and spatiotemporal variables.

A year into the pandemic, protective health behaviours among Uruguayans decreased while mobility increased, according to Google *Community mobility reports*.^10^ In January 2021, as reported by the advisory group, the epidemic in Uruguay had reached the community-transmission level, with a high incidence of cases across the territory. The scientific group therefore proposed a set of actions to curb the advance of the epidemic. The recommendations of the scientific team were, among others, to promote the reduction of mobility, interact mainly with cohabitants, maintain physical distance, ventilate closed areas and use face masks in open spaces. The report, widely disseminated in the media, pointed out that several of the proposed measures were not being implemented at that time. The report also recommended that communications should be adapted to the different target population groups.^11^

The government used the concept of the bubble to recommend protective behaviours: restricted social life and keeping interactions within close family members or one small exclusive group. Nevertheless, the recommendation was not fully understood nor accepted by people, and public communication campaigns were ambiguous and not clear enough.

The data the observatory collected in February and March showed that most people were not willing to cancel small gatherings with friends or other non-cohabiting family members. The bubble concept was not communicated clearly enough to prevent risky behaviours and it did not contribute to the generation of a risk perception matching the epidemiological situation.

Evidence allowed us to identify different behaviours in the population according to their socioeconomic status, gender and age group. We observed that the more severe the epidemiological situation, the higher the risk perception, as was the perception of the effectiveness of some non-pharmaceutical interventions related to reduction of mobility.

The results were widely disseminated in the media, reaching different audiences such as policy-makers, health-care professionals and the general public. The evidence contributed to promoting a public discussion on the behavioural dynamics of the Uruguayan population. The observatory thus played a key role in generating debate on the promotion of non-pharmaceutical interventions and protective health behaviours.

This experience shows the relevance of producing opportune and useful data, which were previously unavailable in the country. 

At least four lessons can be highlighted from this experience. First, integrating behavioural and social data into the pandemic response is a complex process despite the quality of the evidence generated. The translation of knowledge into actions requires greater political and social legitimization and recognition of the relevance of evidence-based interventions and of scientific contribution. We have demonstrated the need to develop tools and communication skills to contribute to the social use of knowledge. Second, the observatory had to negotiate between quality and timeliness of data collection; both are essential in an emergency, but difficult to reconcile. The observatory also had to manage the analysis’ timeline and communication to different audiences in an increasingly unfavourable epidemiological context. Third, the evidence generated was not fully used to develop communication strategies to ensure social and individual protective behaviours in the short-term. We must therefore note the complex relationship between science and policy in the decision-making process, including the limitations on, as well as the possibilities for, the use of knowledge. Fourth, the discussion and political controversies about how to manage the pandemic created a context of tension regarding the role of scientific advice in the process, which also needs to be considered.

During the pandemic, behavioural and social sciences were needed to contribute to the understanding of an uncertain and stressful time for humanity. In Uruguay, the experience of the observatory shows the strengths and limitations of encouraging evidence-based interventions to promote protective health behaviours.
